# Reliability of colour and hardness clinical examinations in detecting dentine caries severity: a systematic review and meta-analysis

**DOI:** 10.1038/s41598-019-41270-6

**Published:** 2019-04-25

**Authors:** Larry Hon, Ahmed Mohamed, Edward Lynch

**Affiliations:** 1 Pediatric Dental Resident, Pediatric Dentistry Department, University of Nevada, Las Vegas, UNLV 89106 USA; 2 Visiting Faculty, Biomedical and Clinical Research, University of Nevada, Las Vegas, UNLV 89106 USA; 3 Professor and Principal Director of Biomedical and Clinical Research, University of Nevada, Las Vegas, UNLV 89106 USA

**Keywords:** Caries risk assessment, Oral diagnosis

## Abstract

Dental caries is the most common human infectious disease and is caused by microorganisms producing acids, resulting in changes in dental tissue hardness and colour. However, the accuracy and reliability of dentine colour and hardness as indicators for carious lesion severity has never been assessed in a systematic review. By applying strict criteria, only seven papers (five randomized control trials and two diagnostic studies) were considered for full text qualitative and quantitative assessment. Only three studies produced high quality evidence and only four articles were considered for meta-analysis, as these provided log_10_ colony forming units (CFU) data from caries biopsies following colour and hardness clinical examinations. When comparing the amount of CFU isolated from carious biopsies from different colour and hardness categories, hardness clinical examination was found to be a statistically more discriminate test than colour clinical examination. Therefore, hardness clinical examination is more specific and reliable than colour to detect dentine carious lesion severity. Further large carefully designed clinical studies are needed to consolidate the findings of this systematic review.

## Introduction

Dental caries is the most common chronic disease amongst all oral conditions^1^. Caries experience is found in 21% of children between the ages of 6–11 years, and in 91% of adults older than 20 years of age in the United States^2^. While odds for untreated decay increases with decreasing income, dental caries prevalence in the primary dentition is 10.8% in the Philippines, 64.2% in India, and 30% in Spain, signifying the varying effects of geographic location and socioeconomic status on onset and spread of dental caries^3–6^.

Demineralized tooth structure and the formation of dental caries is associated with acid production by microbial metabolism of sugar^7^. Studies have shown that the initial colonizing microorganisms are mainly *Streptococcus sanguinis*, *Streptococcus oralis* and *Streptococcus mitis*^8^. Eventually, *Streptococcus mutans* proportionally increases when compared to other decay bacterial species, while surface enamel changes to a frosty white colour caused by the acid damage^8^. This domination of S. *mutans* will subsequently reduce at the advancing stages of dental caries due to the increasing numbers of other microorganisms, such as *Lactobacilli*, *Prevotella*, and *Bifidobacterium*^9^. Therefore, the detection of microorganisms and the number of microbial colonies formed inside carious lesions can be considered as definitive and sensitive indicators for carious lesion severity. Carious lesion severity could be essentially defined as the level of clinical treatment needs (e.g. intrusive or minimal) of a carious lesion based on the amount of microbial colonies isolated from the carious lesion as dentinal lesions that contain large amounts of isolated microbial colony forming units (CFUs) often require a more intrusive clinical treatment (e.g. caries debridement and restoration) if compared to those with less isolated CFUs which may either require less intrusive treatments such as chemotherapeutic treatments (e.g. topical fluoride treatment) or no treatment need at all^10–14^. Furthermore, by identifying the number and types of microbial species isolated from carious lesions, the stages of caries progression might be distinguished with great specificity, sensitivity, and reliability.

The most common methods used for dental caries detection in clinical practice are visual and tactile examinations^15^. Under normal daylight, the colour of a lesion is categorized by visual comparison with a standard guide of four shades (yellow, light brown, dark brown, and black) which is prepared from photographs of primary dentinal carious lesions. On the other hand, the texture or hardness of lesions are classified into three grades (hard, medium or leathery, soft) as described by Hellyer *et al*.^16^. Briefly, under standard dental lighting, hard lesions are as hard as the surrounding tooth tissue, leathery lesions are penetrated by a new Ash No. 6 probe under modest pressure but displayed resistance to its withdrawal, while soft lesions are easily penetrated by a new Ash No.6 probe under modest pressure and displayed no resistance to withdrawal of the probe^10,17^. However, visual and tactile examinations result in low reproducibility and low sensitivity due to their subjective nature, but produce highly specific outcomes^18^. Studies indicated that cariogenic microorganisms produce acids that destroy tooth structure, resulting in changes of colour, consistency, and moisture content of dental tissue. For instance, darker and softer carious lesions contained larger numbers of microorganisms^10,14,19,20^. Thus, to improve the sensitivity, specificity, and reliability of clinical dentine caries examination detection methods, it is suggested that visual and tactile criteria for caries detection should be assessed in relation to the carious microbial activity as this would provide an accurate consistent method to assess caries quantitatively and qualitatively.

Therefore, the aim of this study was to systematically review the literature into colour and hardness of dentine caries and their association with microbial activity, particularly the amount of microbial colony forming units (CFU) that are isolated clinically from biopsies of these lesions following colour and hardness clinical examinations. This potentially proves which of the categories of colour or hardness is more specific and reliable to reflect the severity of carious lesions.

## Methods

The Preferred Reporting Items for Systematic reviews and Meta-Analysis (PRISMA) guidelines were adopted for the current study^21^.

### Search strategy

Studies that assessed the association between colour and hardness with the quantification of CFUs from biopsies of these carious lesions as an indicator of dentinal carious lesion severity were accessed using a defined search strategy in the following electronic databases from 1950 to April 1st, 2018: PubMed, Medline via Ovid, and Web of Science. Handsearching was also performed by accessing the following journals: Community Dentistry and Oral Epidemiology, Caries Research, Journal of Dental Research, Journal of Pediatric Dentistry, Journal of Dentistry, Journal of Oral Health and Preventive Dentistry, and Journal of Dentistry for Children to April 1^st^, 2018. The following search terms were used: “color” or “colour” or “hardness” or “texture” or “consistency” and “dentin caries” or “dentine caries” and “microbiology” or “microflora” or “bacteria”.

### Eligibility criteria

**Figure 1:**
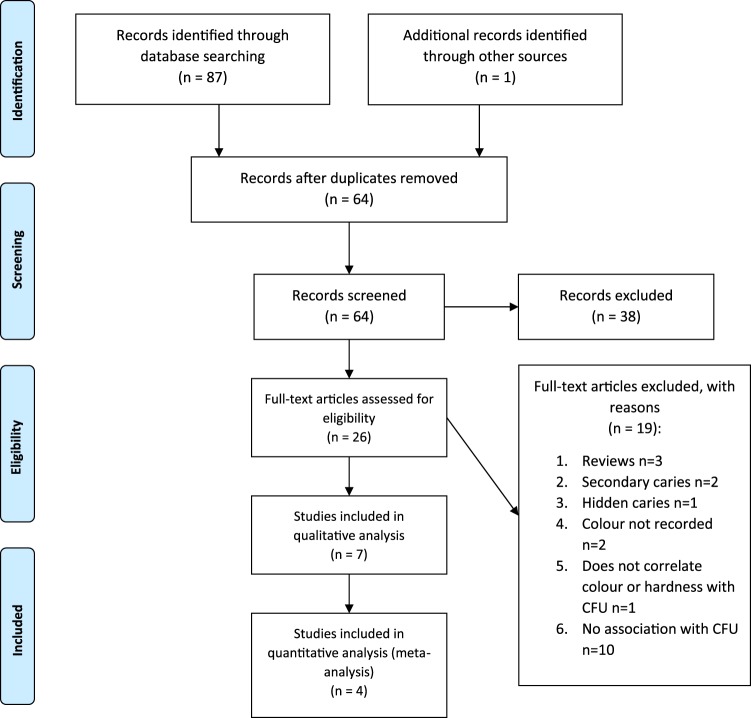
Flow diagram of study selection process.

Search strategy and literature search findings were reviewed by two authors (Hon and Mohamed) to determine whether the identified studies met the inclusion criteria. The inclusion criteria were as follows: (1) randomized controlled trial (RCT) or diagnostic tests studies; (2) colour and hardness scored clinically; (3) CFU reported; (4) dentine caries; (5) open primary dentine caries in deciduous or permanent teeth; (6) articles published in English; and (7) human studies *in vivo*. Animal studies, *in vitro* studies, reviews, comments, editorials, and non-English studies were excluded. Additionally, research that contained irreversible pulpitis, secondary caries, hidden caries or caries that could only be seen radiographically were excluded. Disagreements between the two authors were resolved by an independent reviewer (Lynch). For detailed information, please refer to Fig. 1.

### Data extraction and quality assessment

**Figure 2:**
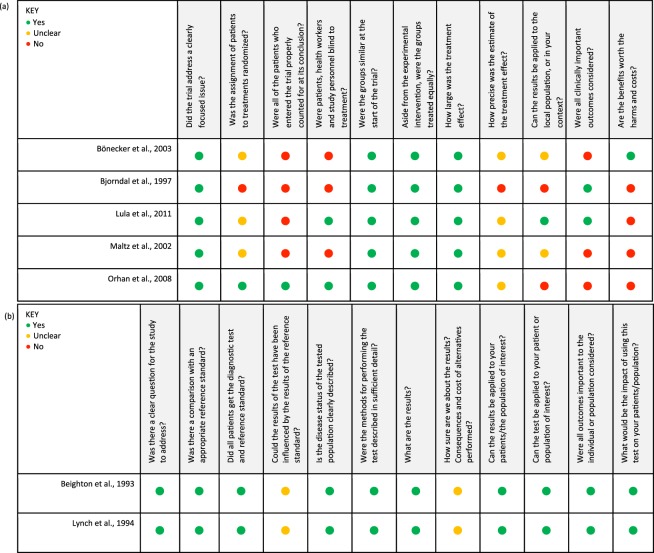
Qualitative analysis with CASP tools for randomized controlled trials. Summary review of the qualitative assessment of the included studies by using CASP tools for randomized controlled trials (RCTs) consisting of 11 quality criteria (**a**) and for diagnostic studies consisting of 12 quality criteria (**b**). Green-coded circle indicates that the study satisfactorily met the respective quality criterion, yellow-coded circle indicates that the study partially met the respective quality criterion, and the red-coded circle indicates that the study did not meet the respective quality criterion.

Two authors (Hon and Mohamed) extracted data using a pre-set data extraction sheet. The data extraction sheet included the following information: author, title, population, intervention, comparison, sample size, confidence level, results and outcomes. For the data extraction details, please refer to Table 1. The quality of evidence in the included studies was assessed by the Critical Appraisal Skills Programme (CASP) tools^22,23^. The included studies were randomized controlled trials and diagnostic tests studies. Thus, the respective CASP tools were used to assess each study type. Each article was independently assessed by two authors (Hon and Mohamed), while disagreements were resolved by an independent reviewer (Lynch). Inter-examiner variability among the 2 examiners was measured by calculating the percentage of agreement (%) among the 2 examiners in each CASP checklist criteria. This is due to the low number of included studies (diagnostic studies n = 2 and RCTs n = 5)^24–27^. For detailed information, please refer to Fig. 2.

**Table 1:** Studies used for qualitative analysis.

Study	Population	Intervention	Comparison	Outcome of Interest	Sample Size	Confidence Level	Results and Findings
Orhan *et al*.	154 teeth	One-visit Indirect pulp treatment (IPT).	Two-visit IPT, and direct complete excavation (DCE)	CFU (CFU/ml)	No sample size calculations.	Confidence level was reported.	Lesions classified by colour did not show difference in CFU of bacteria (p > 0.05), but harder lesions showed less CFU of bacteria than softer lesions (p < 0.05).
Bjørndal *et al*.	31 teeth	Step-wise excavation	Paired study design	CFU count	No sample size calculations.	Confidence level was not reported.	No statistical data was provided.
Maltz *et al*.	32 teeth	Step-wise excavation	Paired study design	CFU (log_10_(cfu + 1))	No sample size calculations.	Confidence level was reported.	Authors did not statistically analyze and compare CFU of hardness and colour categories.
Bönecker *et al*.	40 teeth	ART removal of carious dentin and immediately restored with Glass Ionomer.	Paired study design	CFU (CFU/ml × 10^3^)	No sample size calculations.	Confidence level was reported.	No statistical significant difference results reported.
Lula *et al*.	16 teeth	Removal of carious dentin; only a superficial layer of carious dentin was removed from the pulpal wall.	Paired study design	CFU (log_10_ (CFU/mg))	Sample size was calculated based on pilot study with 6 teeth (80% power test at 5% level of significance)	Confidence level was reported.	No difference in bacteria counts between colour categories. Also, no difference in bacteria counts between hardness categories. Authors did not compare bacteria count of colour to hardness.
**Author**	**Population**	**Exposure**	**Comparison**	**Outcome of Interest**	**Sample Size**	**Confidence Level**	**Results and Findings**
Lynch *et al*.	117 patients	Caries examination by colour	Caries examination by texture	CFU (log_10_ (CFU + 1))	No sample size calculations.	Confidence level was reported.	Black soft and black leathery lesions had higher CFU counts. Soft and leathery lesions generally had a higher count in microorganisms regardless of color. There was no indication that lesion color was significantly associated with total bacteria or proportions of species.
Beighton *et al*.	59 patients	Caries examination by colour	Caries examination by texture	CFU (log_10_ (CFU + 1))	No sample size calculations.	Confidence level was reported.	Soft lesions contained significantly (p < 0.001) greater CFU than leathery lesions, and samples from leathery lesions contain greater CFU (p < 0.001) than hard lesions.

### Statistical analysis

Clinical recordings using colour and hardness of carious dentine have been proposed as indicators of lesion severity. Microbiological biopsies of carious samples have been used to enumerate the numbers of microorganisms expressed as CFUs. The primary outcome measures analyzed were: total microbial load in CFU in each category of colour (yellow, light brown, dark brown, black) and hardness classification (soft, medium hard or leathery, hard). Differences (d_colour_) in means of total microbial load between categories of the colour scale were assumed as a measure of “discriminant power” using this test. Three differences between adjacent categories were computed as: yellow vs. rest, yellow/light brown vs. dark brown/black, black vs. rest. Differences (d_hardness_) in means of total microbial load between categories of the hardness scale were also assumed as a measure of “discriminant power” using this test: hard vs. rest, soft vs. rest. If the difference obtained from the colour test is significantly higher than those from the hardness test, it means that the colour test is more discriminating than the hardness test. Inversely, if the difference obtained from the colour test is significantly lower than those from the hardness test, it means that the colour test is less discriminating than the hardness test. Therefore, mean differences between all colour and hardness category differences were computed and the weighted mean difference (WMD) was the global effect measure in a random-effects model.

In addition, the effect size of the differences between colour (or hardness) categories regarding mean CFU was also calculated. The effect size index for a conventional one-way ANOVA is…$f=\sqrt{\frac{{{\beth }}^{2}}{1-{{\beth }}^{2}}}$, whereby ${\beth }^{2}$ is the ratio of the between-groups variance to the total variance. The larger the effect size “*f* ” is, the larger the difference between mean total microbial load of categories is, meaning there is a higher power to discriminate. As a general convention, small $f=0.10$, medium $f=0.25$, and large $f=\mathrm{0.40.}\,$An overall effect size was estimated weighting the individual numbers by the sample size of each study. The level of significance used in the analysis was 5% (α = 0.05). The software used to perform this meta-analysis was R 3.0.2 and its ‘metafor’ package. The software Gpower 3.1.3 was used to estimate effect sizes.

## Results

### Study characteristics and quality assessment

Fig. 1 shows the detailed steps used for the literature search. Of the 64 potentially relevant articles, 26 articles were eligible for full text screening. However, 19 articles were further excluded because they did not meet the inclusion criteria. Please refer to Table 2 for detailed information on the excluded papers and the rationale for their exclusion. Therefore, only seven articles^10,14,19,28–31^ were included for full text quantitative and qualitative assessment in this systematic review. These seven articles were published between 1950 and 2018. Among these articles five were randomized controlled trials^19,28–31^ and two were diagnostic test studies^10,14^.

**Table 2:** Excluded studies and rationale for exclusion.

Study	Reason for exclusion
Kidd *et al*.^34^	Colour not recorded
Weerheijm *et al*.^35^	Hidden caries, not gross occlusal caries
Kidd *et al*.^36^	Includes secondary caries
Ayna *et al*.^20^	No results correlating hardness and CFU
Loesche *et al*.^37^	Incipient caries
Manji *et al*.^38^	No CFU
Bönecker *et al*.^39^	No CFU
Iwami *et al*.^40^	No CFU
Fusayama *et al*.^41^	No CFU
Iwami *et al*.^42^	No CFU
Torii *et al*.^43^	No CFU
Iwami *et al*.^44^	No CFU
Nyvad *et al*.^45^	No CFU
Milnes *et al*.^46^	No CFU
Nyvad *et al*.^47^	No CFU
Fejerskov *et al*.^48^	Review Article
Kidd *et al*.^49^	Review Article
Takashashi *et al*.^50^	Review Article
Kidd *et al*.^51^	Secondary Caries

### A-Randomized controlled trials

Five randomized controlled trial articles were included^19,28–31^. One article disclosed the gender of the participants: 51 females and 60 males^28^. Three articles detailed the age of participants age: 4–15 years old^28^, 12–23 years old^29^, and 5–8 years old^31^; therefore, the range of the age groups of the participants within the latter three studies was 4–23 years old. Three articles specified the type of teeth that received intervention and comparator treatments: 94 mandibular second primary molars and 60 mandibular first permanent molars^28^ and primary molars^30,31^. Only one article provided the number of subjects recruited^28^, whilst the other studies only reported the number of teeth investigated^29–31^. Sample size calculations were only carried out by Lula *et al*., who conducted sample size calculations based on a pilot study where 16 teeth obtained an 80% power at a 5% statistical significance. It was noticeable that the article by Bjorndal *et al*. did not give details as to the gender, age, or type of teeth used (incisor, canine, premolars, molars, primary dentition, and permanent dentition).

As we investigated the intervention and comparison groups, colour and hardness of lesions were related to the total CFU each contained. Two articles scored colour and hardness of lesions whilst evaluating different levels of carious dentine prior to final restoration in a stepwise approach^19,28^, which was using incomplete removal of dentine caries to try to prevent pulpal exposure. Three articles related total CFU in carious dentine with colour and hardness at different time points, at intervals of 6–7 months^29^, 4–6 months^30^, and 3–6 months^31^. Refer to Table 1 for further details on study characteristics. The CASP quality assessment for these included randomized controlled trials is presented in Fig. 2. According to the CASP analysis, the study of Lula *et al*. produced the highest quality of evidence and therefore its findings carried more weight. The inter-examiner variability for quality assessment was 72.7% for the five RCT’s, signifying high reproducibility between the two examiners.

### B-Diagnostic test studies

Two articles were diagnostic test studies^10,14^. Both articles examined the colour and hardness of carious lesions and compared these to their respective total CFU counts^10,14^. Both these articles reported the number of participants and gender**:** 45 females and 72 males^14^, 25 females and 34 males^10^. Therefore, the total number of participants in each gender was 70 females and 106 males. Both articles examined the same age range: 29–80 years old^10,14^. Both articles^10,14^ reported the number of teeth included in each study but neither article detailed the type of teeth that had been examined (incisor, canine, premolars, molars, primary dentition, permanent dentition). Neither article reported any sample size calculation^10,14^. For further details on study characteristics, please refer to Table 1. According to the CASP quality assessment, both articles produced equally high-quality evidence. For further detail CASP analysis of the aforementioned articles, please refer to Fig. 2. The inter-examiner variability for quality assessment was 83.3% for the diagnostic test studies, signifying high reproducibility between the two examiners.

## Comparison of CFU’s Between Colour and Hardness Examination Methods

### A-Randomized controlled trials

One article compared CFUs in one-visit indirect pulp treatment (IPT) with two-visit IPT and direct complete excavation (DCE)^28^. Four articles were paired study designs that compared samples from different time points^19,29–31^. These four articles measured the CFUs taken for colour or hardness categories. The total CFU counts were only measured and compared within the categories of caries colour or categories of caries hardness independently^19,28–30^. Only one article related both colour and hardness to total CFU. This study also compared the CFU counts within each caries colour category (i.e. yellow, light brown, dark brown) and within caries hardness categories (i.e. soft, medium hard, hard)^31^.

Findings of statistical significance are summarized in the study characteristic Table 1. Two articles^28,31^ found no statistical significance in total CFU, S. *mutans* or for *Lactobacillus* spp., recovered from different colour categories but harder lesions contained less total CFU than softer lesions^28,31^. One article did not carry out any statistical analysis between the microflora associations with colour and/or hardness categories because the study did not include sufficient number of samples^30^.

### B-Diagnostic test studies

Both articles assessed the relationships between total CFU with colour and hardness^10,14^. Total CFUs was highest in all soft colour categories compared to all colour categories of leathery lesions which in turn contained more total CFU than all hard colour categories^10,14^, and black soft lesions contained more lactobacilli than black leathery lesions^10^. For further details, please refer to Table 1.

### Statistical analysis for pooled CFU’s outcomes

Some of the included papers had to be excluded from the meta-analysis because they did not have a mean load for each category^28^, where microbial levels were only measured by turbidity methods^28,30^, or where data were only shown as percentages of specific species instead of the total CFU^14^.

The microbial load in each colour and hardness category (Black [B], Dark Brown [DB], Light Brown [LB], Yellow [Y], Soft [S], Medium [M], Hard [H]) was calculated by measuring the log_10_CFU in the studies by Bjorndal *et al*., Maltz *et al*., Lula *et al*., and Beighton *et al*. as shown in Table 3. When lesions were described as leathery these were classified as medium for this analysis.

**Table 3:** Shows the initial summary data in the included studies: means (sd).

Author		Yellow	Light brown	Dark brown	Black	Soft	Medium-hard (leathery)	Hard
Orhan 2008	CFU/ml							
Bjorndal 1997	log10 (CFU)			2.70 (1.24)	2.97 (0.93)		2.59 (1.07)	3.23 (1.28)
Maltz 2002	log10 (CFU + 1)	4.50 (0)	4.07 (0.93)	4.25 (0.71)		4.17 (0.84)		
Bonecker	CFU/ml × 10^3^							
Lula 2011	log10 (CFU/mg)	4.77 (0.32)	2.29 (2.97)	2.66 (3.08)		4.49 (3.00)	2.50 (2.65)	0.00 (0)
Lynch 1994	*Percentages*							
Beighton 1993	log10 (CFU)	4.60 (2.22)	3.50 (1.81)	3.40 (1.89)	6.00 (0.99)	6.80 (0.37)	4.30 (0.56)	1.80 (0.82)

**Figure 3:**
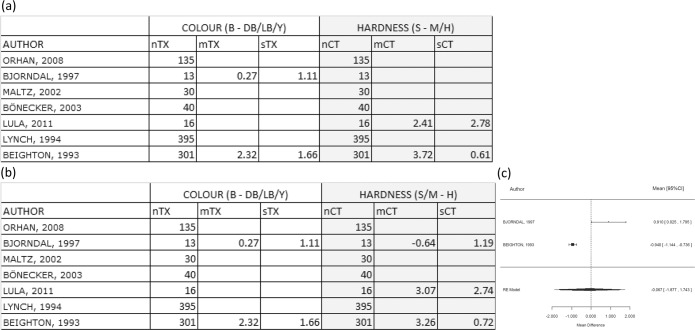
Comparison B-DB/LB/Y versus S-M/H and S/M-H. Comparing mean differences for log(CFU) between categories B-DB/LB/Y (colour test) and categories S-M/H (hardness test) (**a**) and comparison between black against other colour categories with hard and other hardness categories (**b**,**c**). (**b**) Table shows mean differences for log(CFU) between categories B-DB/LB/Y (colour test) and categories S/M-H (hardness test). (**c**) Forest plot showing WMD value for the difference is −0.067. The effect size on population is estimated between −1.88 and 1.74 with a CI of 95%. It should be noted that the interval includes zero, so no significant difference was reached (p = 0.942). TX = treatment/exposure group (colour test); CT = control group (hardness test); n = number of samples; m = mean; s = standard deviation.

When comparing B-DB/LB/Y vs. S-M/H, the only possible comparison was by using the study by Beighton *et al*. It was found that there were more CFU (2.32 units of log_10_ CFU in average) in black samples than any of the other colour groups. In addition, it was found that there were on average 3.72 units more of log_10_CFU in soft samples than in leathery samples and in turn these had significantly more CFU than in hard lesions. The differences in CFU between the hardness categories were considerably more than any differences between the CFU contained in the different colour categories. Therefore, the hardness test was more discriminant, but no meta-analysis was conducted because only 1 study was involved in this comparison as shown in Fig. 3.

When comparing B-DB/LB/Y vs. S/M – H, Beighton *et al*. and Bjorndal *et al*. provided data in relation to CFU differences between these categories, so a meta-analysis was performed, and a forest plot is shown in Fig. 3. The WMD value for the difference was −0.067. The effect size on the population was estimated to be between −1.88 and 1.74 with a confidence of 95%. It should be noted that the interval includes zero, so no significant difference was reached (p = 0.942). Therefore, none of these tests were more discriminant than the other.

**Figure 4:**
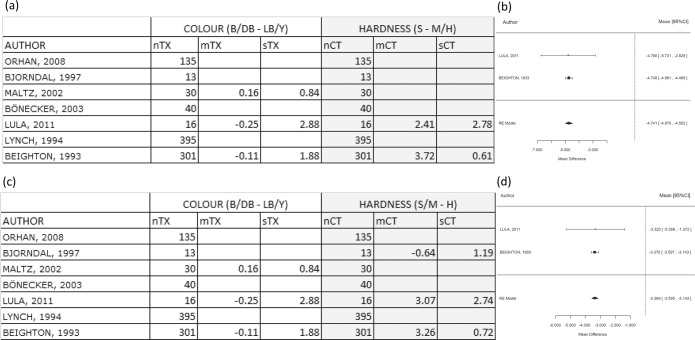
Comparison B/DB - LB/Y versus S - M/H and comparison B/DB - LB/Y versus S/M - H. Comparison between dark brown/black against other yellow/light brown with soft and other hardness categories (**a**,**b**) and comparison between dark brown/black against other yellow/light brown with hard and other hardness categories (**c**,**d**). (**a**) Table shows mean differences for log(CFU) between categories B/DB - LB/Y (colour test) and categories S-M/H (hardness test). (**b**) WMD value for the difference is −3.67 (p < 0.001), favoring the hypothesis that the hardness test is more discriminant. (**c**) Table shows mean differences for log(CFU) between categories B/DB - LB/Y (colour test) and categories S/M - H (hardness test). (**d**) WMD value for the difference is −3.37 (p < 0.001), favoring the hypothesis that the hardness test is more discriminant. TX = treatment/exposure group (colour test); CT = control group (hardness test); n = number of samples; m = mean; s = standard deviation.

Because of the large sample size of Beighton *et al*., (n = 301) in the comparisons between B/DB - LB/Y vs. S - M/H, its weight was very dominant compared to Lula *et al*. (n=16). The overall effect measure of the meta-analysis was similar to the Beighton *et al*. individual results. The WMD value for the difference was −3.67 (p < 0.001), indicating that the hardness test was more discriminant than colour. The analysis for this forest plot is shown in Fig. 4.

When comparing B/DB - LB/Y vs. S/M – H, the results are similar to the previous comparison. The WMD value for the difference was −3.37 (p < 0.001), suggesting that the hardness test was again more discriminant than colour and the forest plot of this analysis is presented in Fig. 4.

**Figure 5:**
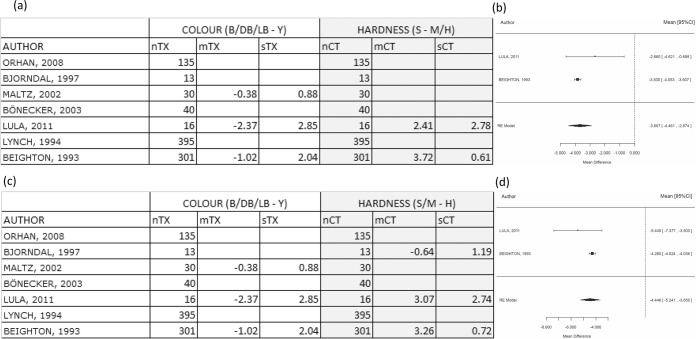
Comparison B/DB/LB - Y versus S - M/H and comparison B/DB/LB - Y versus S/M - H. Comparison between yellow against other colours with soft and other hardness categories. (**a**) Table shows mean differences for log(CFU) between categories B/DB/LB - Y (colour test) and categories S - M/H (hardness test). (**b**) WMD value for the difference is −4.74 (p < 0.001), favoring the hypothesis that the hardness test is more discriminant. Comparison between yellow against other colour with hard and other hardness categories. (**c**) Table shows mean differences for log(CFU) between categories B/DB/LB - Y (colour test) and categories S/M - H (hardness test). (**d**) WMD value for the difference is −4.45 (p < 0.001), favoring the hypothesis that the hardness test is more discriminant. TX = treatment group (colour test); CT = control group (hardness test); n = number of samples; m = mean; s = standard deviation.

When comparing B/DB/LB - Y vs. S - M/H, the WMD value for the difference was −4.74 (p < 0.001), supporting that the hardness test was again more discriminate than colour. The analysis for this forest plot is shown in Fig. 5.

When comparing B/DB/LB - Y vs. S/M – H, the WMD value for the difference was −4.45 (p < 0.001), also indicating that the hardness test was more discriminant than colour. The forest plot of this analysis is shown in Fig. 5.

For Bjorndal *et al*., Lula *et al*. and Beighton *et al*. the effect size for the hardness category is larger than the colour categories; for Bönecker *et al*. the results again favored hardness over colour. Consequently, the weighted total effect size showed a large advantage for hardness as a discriminant rather than colour. However, this difference is caused, especially, by the huge dominance of the Beighton *et al*. data in the overall numbers. The effect size table is shown in Table 4.

**Table 4:** Summary of the effect size (f) of the multiple comparisons of bacterial load means in colour and hardness examinations.

	Colour test	Hardness test
**ORHAN**, **2008**		
BJORNDAL, 1997	0.10	0.28
MALTZ, 2002	0.05	—
BÖNECKER, 2003^a^	0.26	0.23
LULA, 2011	0.38	0.69
**LYNCH**, **1994**		
BEIGHTON, 1993	0.55	3.14
**Total effect size**	0.46	2.62

Effect size (f) for a one-way analysis of variance to estimate differences in total bacteria load between categories of the colour and hardness test. Total effect size is the weighted (by sample size) mean from the authors. It is possible to estimate the effect size for Bönecker because it does not depend on the original units. The effect size is a standardized value.

## Discussion

Dental caries involve microorganisms excreting acid resulting in changes in dental tissue hardness and colour. However, the accuracy and reliability of dentine colour and hardness as indicators for carious lesion severity has never been assessed in a systematic review. Thus, the aim of this study was to systematically analyze published research investigating whether colour or hardness of dentine caries was a more accurate, reliable, and valid method in detecting carious lesion severity when related to the amount of detectable CFU in biopsies of these lesion categories.

Seven papers met the inclusion criteria for this systematic review from searching multiple electronic databases and hand searching multiple journals: five articles were RCTs and two were diagnostic test studies. The five included RCTs were not primarily conducted to investigate the relationship between colour and hardness with the numbers of microorganisms. They were rather trial studies to investigate certain clinical interventions (e.g. one-visit IPT, two-visit IPT, DCE, step-wise excavation, and atraumatic restorative treatment). For the purpose of this systematic review, only the RCT data related to the CFU counts obtained from carious biopsies following the colour and hardness measurements were reported.

The quality of each study was critically appraised using CASP tools. The RCT and diagnostic test studies have separate CASP tools to systematically examine and appraise the evidences of each article. Using CASP tools, it was noticeable that many articles lacked details of patient’s age, gender, type of teeth, socioeconomic status, ethnicity, diet, or sample size calculations^19,30,31^. This would question the quality of evidence in the included papers. In fact, the unreported randomization protocols^10,14,19,29–31^, sample size calculations^10,14,19,28–30^, and confidence limits^10,14,19,28–31^ are additional evidence of the low quality of evidence in most of the included studies. This study shows that only the studies by Lula *et al*., Lynch *et al*. and Beighton *et al*. reported acceptable levels of evidence.

From the meta-analysis, only 4 articles were considered for meta-analysis, as these provided CFU data for different categories of colour and hardness. Differences in log_10_CFU between categories of hardness were significantly greater than between categories of colour. However, CFU results were also not consistently reported. For instance, all 30 samples for Maltz *et al*. were categorized as ‘soft’, thus no comparison was possible. Also, Bjorndal *et al*. provided CFU for samples (n = 31) but they all were classified exclusively in only 2 colour categories (dark brown and black) and 2 hardness categories (medium hard and hard). Therefore, comparisons had to be focused to results from Lula *et al*. and Beighton *et al*. The difference between their sample sizes was extreme, so overall results from the meta-analysis inherited mostly properties and results from Beighton *et al*. As with weighted effect size calculations, results data also showed a large advantage for hardness examination compared to colour examination. However, unlike pooled meta-analysis calculations, the study by Bönecker *et al*. was added to the weighted total effect size calculations. This was possible because effect size can be calculated regardless of the differences in the reported units of CFUs as effect size calculations measure ratio of variability between groups to within-groups^32^. Nevertheless, the meta-analyses and effect size calculations data in this study indicate that the texture (or hardness) tactile clinical examination has more discriminatory power in comparison to the colour visual clinical examination. Therefore, hardness or texture categories are more reliable in reflecting the amount of isolated cariogenic microorganisms and thereby more reliable in detecting the severity of carious lesions. Thus, based on the findings of this study, the hardness tactile clinical examination is a more specific and sensitive clinical examination method as hardness categories would be more reliable indicators for treatment planning in which soft carious lesions would harbor more cariogenic microorganisms, if compared to hard carious lesions, and therefore may require a more intrusive dental clinical intervention. These findings are consistent with previously reported data in which hard carious lesions were found to harbour no cariogenic bacteria (e.g. neither streptococci nor lactobacilli) and therefore required no dental clinical interventions. On the other hand, a large proportion of soft carious lesions contained the latter species (63.6% and 48.4% respectively) and required both caries debridement and restoration^10^.

The findings of this systematic review can be viewed as additional evidence in support of the International and Caries Detection Assessment System (ICDAS), which is currently considered as the recommended dental caries examination scoring index in dental practice^33^. In this study, hardness clinical examination was found to be more specific and reliable than colour examination. Thus, changes in dental tissue hardness should warrant more scores in dental caries classification indices, which match the current setup of the ICDAS that emphasizes on the importance of hardness clinical examination by allocating more scores to changes in hardness^33^.

The findings of this systematic review must be interpreted with caution as there are a number of limitations. Firstly, it was not possible to group study data and findings in all of the included 7 studies due to the inconsistency and heterogeneous approaches in which the authors recorded teeth colour, hardness, and CFU. For instance, the articles reported various methodologies for detecting and analyzing microbial CFU. Also, the authors used dissimilar approaches to report CFU data, such as: some studies presented the results with “total CFU counts”^19,28^, while other studies used “turbidity tests”^28,30^. Other articles normalized the CFU data by using log_**10**_(CFU/mg)^31^ or log_10_(CFU + 1)^10,14,29^. Secondly, all data tables presented by the diagnostic test studies^10,14^ showed CFU percentages of different bacterial species instead of stating the exact numerical actual counts, which further made it difficult to group and compare data and findings across the studies and therefore made it difficult for conducting a meta-analysis of all included studies which also hampered our ability to have an overall systematic understanding of the data. Finally, most of the included studies were RCTs, a design that is more vulnerable to sampling bias without accurate sample size calculations. It was noticeable in this systematic review that sample size calculation was only reported in one study^31^.

In summary, this study presents systematically reviewed evidence in support of hardness clinical examination being a more reliable and specific dental caries detection method. Therefore, it is recommended to consider the use of tactile examination over visual inspection for caries detection during routine and treatment dental visits. However, due to the limitations in this study, further research is needed to consolidate the findings in this systematic review.

In conclusion, the microbial differences between hardness types showed a weighted total effect size with a large advantage for hardness compared to colour and colour alone also yielded inconsistent microbial results. Hardness is more reliable than colour to detect dentine caries severity.
